# The crop mined phosphorus nutrition via modifying root traits and rhizosphere micro‐food web to meet the increased growth demand under elevated CO_2_

**DOI:** 10.1002/imt2.245

**Published:** 2024-10-25

**Authors:** Na Zhou, Xue Han, Ning Hu, Shuo Han, Meng Yuan, Zhongfang Li, Sujuan Wang, Yingchun Li, Hongbo Li, Zed Rengel, Yuji Jiang, Yilai Lou

**Affiliations:** ^1^ Institute of Environment and Sustainable Development in Agriculture, Chinese Academy of Agricultural Sciences Beijing China; ^2^ School of Food and Biological Engineering Hezhou University Hezhou China; ^3^ Soil Science & Plant Nutrition, UWA School of Agriculture and Environment The University of Western Australia Perth Australia; ^4^ College of Resources and Environment Fujian Agriculture and Forestry University Fuzhou China

**Keywords:** AMF, co‐occurrence network, microbiome, micro‐food web, nematode, P fraction, root exudation

## Abstract

Elevated CO_2_ (eCO_2_) stimulates productivity and nutrient demand of crops. Thus, comprehensively understanding the crop phosphorus (P) acquisition strategy is critical for sustaining agriculture to combat climate changes. Here, wheat (*Triticum aestivum* L) was planted in field in the eCO_2_ (550 µmol mol^−1^) and ambient CO_2_ (aCO_2_, 415 µmol mol^−1^) environments. We assessed the soil P fractions, root morphological and physiological traits and multitrophic microbiota [including arbuscular mycorrhizal fungi (AMF), alkaline phosphomonoesterase (ALP)‐producing bacteria, protozoa, and bacterivorous and fungivorous nematodes] in the rhizosphere and their trophic interactions at jointing stage of wheat. Compared with aCO_2_, significant 20.2% higher shoot biomass and 26.8% total P accumulation of wheat occurred under eCO_2_. The eCO_2_ promoted wheat root length and AMF hyphal biomass, and increased the concentration of organic acid anions and the ALP activity, which was accompanied by significant decreases in calcium‐bound inorganic P (Ca‐P_i_) (by 16.7%) and moderately labile organic P (by 26.5%) and an increase in available P (by 14.4%) in the rhizosphere soil. The eCO_2_ also increased the growth of ALP‐producing bacteria, protozoa, and bacterivorous and fungivorous nematodes in the rhizosphere, governed their diversity and community composition. In addition, the eCO_2_ strengthened the trophic interactions of microbiota in rhizosphere; specifically, the eCO_2_ promoted the associations between protozoa and ALP‐producing bacteria, between protozoa and AMF, whereas decreased the associations between ALP‐producing bacteria and nematodes. Our findings highlighted the important role of root traits and multitrophic interactions among microbiota in modulating crop P‐acquisition strategies, which could advance our understanding about optimal P management in agriculture systems under global climate changes.

## Highlights

- Elevated CO_2_ (eCO_2_) promoted wheat root length, organic acid anions, arbuscular mycorrhizal fungi (AMF) hyphal biomass, and wheat phosphorus (P) accumulation
- eCO_2_ decreased calcium‐bound inorganic P (Ca‐P_i_) and moderately labile organic P but increased available P in the rhizosphere
- eCO_2_ increased the growth of alkaline phosphomonoesterase (ALP)‐producing bacteria, protozoa and bacterivorous and fungivorous nematodes and strengthened their trophic interactions

## Graphical abstract

The Elevated CO_2_ (eCO_2_) promoted wheat phosphorus (P) accumulation through increased root length and arbuscular mycorrhizal fungi (AMF) hyphal biomass, the concentration of organic acid anions and the alkaline phosphomonoesterase (ALP) activity. The eCO_2_ also increased the growth of ALP‐producing bacteria, protozoa and bacterivorous and fungivorous nematodes in the rhizosphere, and strengthened their trophic interactions.

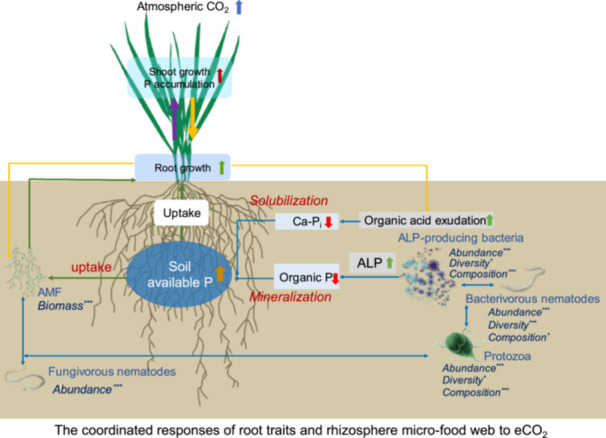

## INTRODUCTION

Phosphorus (P) is essential for growth of plants and soil biota in various environments [1]. Although total P is abundant in many soils, in alkaline soils it is present mostly as the calcium‐bound inorganic P (Ca‐P_i_) or organic P (P_o_) forms [2]. Only small amounts of inorganic P (orthophosphate) are available to plants and microbes in many low‐input agricultural systems, which frequently limits the crop productivity [3]. Plants have evolved several strategies to acquire P to meet their growth demand: (1) increasing root growth to extend root absorptive surface to directly capture labile inorganic soil P [4], (2) strengthening symbiotic interactions with arbuscular mycorrhizal fungi (AMF) to explore a large soil volume and capture labile inorganic soil P via external AMF hyphae [5, 6], (3) releasing more P‐mobilizing exudates (e.g., organic acid anions) into the rhizosphere [3, 7, 8], and (4) stimulating growth of phosphate‐solubilizing bacteria in the rhizosphere to enhance organic P mineralization via phosphatase enzymes [9]. However, how plants adjust or coordinate these strategies to enhance P acquisition under elevated CO_2_ (eCO_2_) remains largely unknown.

Elevated CO_2_ can significantly increase plant biomass [10, 11], especially when the available P in soil is sufficient to meet plant requirements. The increased P removal with crop harvests over years can lead to the depletion of available P under eCO_2_ [12], but soil available P can also be replenished through the mobilization of inorganic P and the mineralization of organic P under eCO_2_ [13, 14]. In addition, under eCO_2_, plants can increase their C allocation to belowground parts to promote root growth [15] as well as root exudation, which can enhance soil P mobilization and provide organic substrates for the growth and metabolic activity of microbiome in the rhizosphere [16, 17]. The P immobilization into microbial biomass can diminish plant capacity to capture available P under eCO_2_ [18]. However, as microbial biomass P decays, remineralization of P may contribute significantly to replenishing available P and promoting crop productivity [19]. In addition, eCO_2_ can stimulate significantly root colonization by AMF [20, 21, 22] and manipulate AMF community composition to increase the efficiency of P capture [23], thereby enhancing plant growth [24].

To date, most studies have focused on abiotic parameters and plant traits influencing the growth and composition of phosphate‐solubilizing bacteria and AMF communities [25, 26], but less attention has been paid to protozoa and nematodes that also play key roles in shaping microbial community structure and nutrient cycling through selective predation in the rhizosphere [27, 28]. Previous studies have found that the predation by bacterivorous and fungivorous nematodes and protozoa can contribute to P cycling and plant growth [29] via modulating biomass, abundance and composition of phosphate‐solubilizing bacteria [30, 31, 32] and AMF [23, 33]. For instance, Zheng et al. [31] revealed that selective predation by nematodes facilitated the competition of bacterial keystone taxa with the connected members, resulting in enhanced diversity of alkaline phosphomonoesterase (ALP)‐producing bacteria and increased ALP activity in the rhizosphere of rapeseed (*Brassica napus* L.) that enhanced P availability. Jiang et al. [33] showed that selective predation by fungivorous nematodes increased plant colonization by AMF, leading to enhanced plant P accumulation and productivity. In summary, predators may stimulate microbial activity and P mineralization, thereby promoting P availability, P acquisition, and plant growth. So far, the ecological mechanisms explaining how different protozoa and nematodes contribute to P cycling and plant growth through grazing on plant growth‐promoting microorganisms (such as ALP‐producing bacteria and AMF) under environmental changes are seldom explored [34], especially under eCO_2_.

In the present study, a 15‐year free‐air‐CO_2_ enrichment (FACE) was performed to assess changes in wheat P acquisition and soil P fractions under eCO_2_ and clarify the linkages among soil microbiota (including ALP‐producing bacteria, AMF, protozoa, and bacterivorous and fungivorous nematodes) in the rhizosphere of wheat. We asked the following three questions: (1) How can eCO_2_ impact wheat growth, plant P accumulation, and P fractionation in wheat rhizosphere? (2) Whether and how do plants coordinate the root morphological and physiological traits and the communities of ALP‐producing bacteria and AMF to capture P under eCO_2_? (3) How and to what extent are protozoa and nematodes linked to their potential prey (ALP‐producing bacteria and AMF community) under different CO_2_ environments? We hypothesized that eCO_2_ would strongly shape the root traits, growth, and community composition of rhizosphere microbiota as well as their trophic interactions, increasing mobilization of inorganic and organic P fractions in the rhizosphere and crop P uptake.

## RESULTS

### The eCO_2_ promoted the wheat P accumulation through boosting growth of roots and AMF, and P availability in rhizosphere

**Figure 1:**
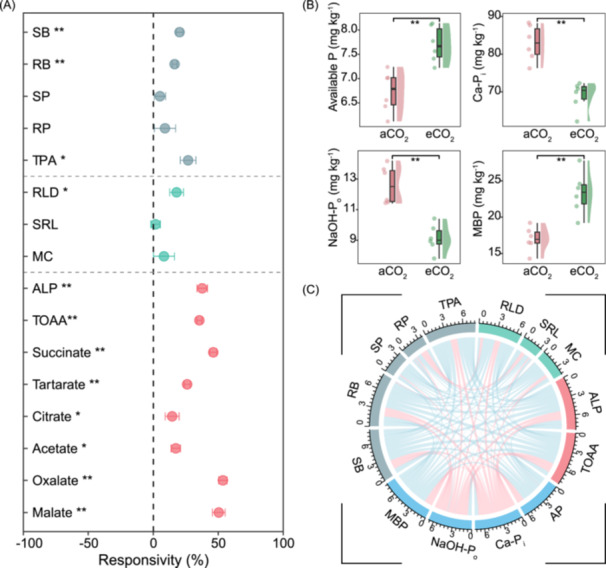
The impact of eCO_2_ on wheat growth, root traits, soil property, and P fraction in the rhizosphere soil. (A) The responsivity of plant growth (SB, RB, SP, RP, TPA), morphological traits (RLD, SRL, MC), and physiological traits (ALP, TOAA, Succinate, Tartarate, Citrate, Acetate, Oxalate, and Malate). (B) The contents of available P (H_2_O‐P_i_ and NaHCO_3_‐P_i_), HCl‐extractable inorganic P (Ca‐P_i_), NaOH‐extractable organic P (NaOH‐P_o_), and microbial biomass phosphorus (MBP) of wheat in the rhizosphere soil under ambient CO_2_ (aCO_2_) and elevated CO_2_ (eCO_2_). The means and standard errors (*n* = 6). (C) The correlation coefficient between plant traits and soil properties and the content of soil P fractions. Pink edges indicate positive and blue edges negative correlations. **p < 0.01; *p < 0.05. ALP, alkaline phosphomonoesterase activity; Ca‐P_i_, HCl‐extractable inorganic P; MBP, microbial biomass P; MC, root mycorrhizal colonization; NaOH‐P_o_, NaOH‐extractable organic P; RB, root biomass; RLD, root length density; RP, root P concentration; SB, shoot biomass; SP, shoot P concentration; SRL, specific root length; TOAA, total organic acid anions (proxy for root exudation); TPA, total plant P accumulation.

Compared to aCO_2_, eCO_2_ significantly increased the shoot biomass (SB, by 20.2%), root biomass (RB, by 16.4%), and total plant P accumulation (TPA, by 26.8%). However, eCO_2_ had no significant effects on the shoot P concentration and root P concentration (Figure 1A). Then, we investigated the influence of eCO_2_ on root morphological and physiological traits. The eCO_2_ increased root length density (RLD) by 17.9%, but specific root length and mycorrhizal colonization did not differ between aCO_2_ and eCO_2_. The eCO_2_ also promoted the release of organic acids (including succinate, tartarate, citrate, acetate, oxalate, and malate), and the concentration of total organic acid anions (TOAA) was increased by 35.4% under eCO_2_ (Figure 1A). In addition, the eCO_2_ significantly promoted ALP activity in the rhizosphere soil (by 37.6%). For the soil P fractions, we found that the eCO_2_ increased available P in the rhizosphere soil by 14.4% but decreased the Ca‐P_i_ fraction by 16.7% and NaOH‐P_o_ by 26.5% (Figure 1B), with no impact on NaOH‐P_i_ and residual P (Figure S1). Further, eCO_2_ decreased the content of total P in the rhizosphere soil by 11.6% (Figure S1). The microbial biomass P (MBP) increased by 38.0% under eCO_2_ (Figure 1B). In the present study, there were positive correlations between wheat P accumulation and RLD, TOAA, ALP activity, and available P, and negative correlations between wheat P accumulation with Ca‐P_i_ and NaOH‐P_o_ (Figures 1C and S2).

**Figure 2:**
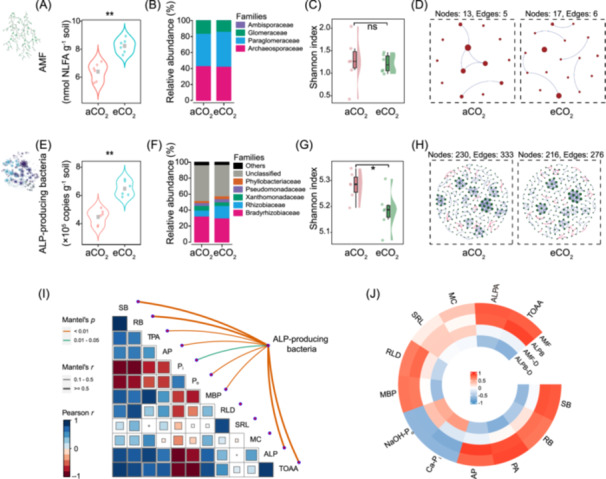
The features of AMF and ALP‐producing bacteria under different CO_2_ treatments. (A–H) The biomass/abundance (A and E), relative abundance of taxa (B and F), diversity (Shannon index) (C and G) and co‐occurrence networks (D and H) of the AMF and ALP‐producing bacteria (ALPB) in the rhizosphere soil under ambient CO_2_ (aCO_2_) and elevated CO_2_ (eCO_2_). **p < 0.01; *p < 0.05. The means and standard errors (*n* = 6). (I) Correlations between the composition of ALP‐producing bacteria community and plant traits and soil properties. Pair‐wise comparisons of those traits are shown, with color gradients denoting Spearman correlation coefficients. Edge width corresponds to the Mantel r statistic for the specific distance correlations. (J) The relationship between the abundance (AMF biomass and ALPB abundance) and the diversity (Shannon index) of soil microbiota communities (AMF‐D, ALPB‐D) and plant traits and soil properties. ns, nonsignificant.

The eCO_2_ also promoted the growth of AMF and ALP‐producing bacteria in rhizosphere. The eCO_2_ treatments significantly increased the biomass of saprophytic fungi (by 28.0%) (Figure S3) and AMF (by 28.9%) (Figure 2A). The eCO_2_ had no impact on the relative abundance of each family and AMF diversity (Figure 2B,C). In the co‐occurrence network for the AMF community, compared with the aCO_2_ treatment (nodes = 13, edges = 5), the node and edge numbers for AMF were higher under eCO_2_ (nodes = 17, edges = 6) (Table S1, Figure 2D). In addition, for AMF the network showed variation in topological metrics, with the lower values of average degree and connectedness in the eCO_2_ compared with aCO_2_ (Table S1). Similarly, compared with the aCO_2_, the treatment with eCO_2_ promoted the biomass of bacteria (by 54.7%, Figure S3), and the abundance of ALP‐producing bacteria by 44.9% (Figure 2E). In addition, the eCO_2_ also promoted the relative abundance of Rhizobiaceae by 105.6% and Phyllobacteriaceae by 51.0% in ALP‐producing bacteria taxa (Figure 2F). In the eCO_2_ treatment, the Shannon index of the ALP‐producing bacterial community decreased by 1.8% (Figure 2G) and the community composition (the first principal coordination [PCo1] scores) of the microbial community changed significantly (Figures 2 and S4). For ALP‐producing bacteria, the eCO_2_ treatment decreased the nodes and edges, whereas the values of average degree decreased slightly, and connectedness did not change (Figure 2H, Table S1).

The community composition of ALP‐producing bacteria had significant correlations with wheat growth (shoot biomass, root biomass, and wheat P accumulation), soil P fractions, ALP activity and concentration of total organic acid anions, whereas the community composition of AMF did not significantly correlate with any plant and soil parameters across the two CO_2_ environments (Figures 2I and S5). The AMF biomass, the abundance of ALP‐producing bacteria and the relative abundance of Rhizobiacea had significant positive correlations with wheat growth, available P, ALP activity, and TOAA exudation, but showed negative correlations with Ca‐P_i_ and NaOH‐P_o_ (Figures 2J and S6). For the diversity, the Shannon index of ALP‐producing bacteria had negatively correlations with shoot biomass, wheat P accumulation, MBP and TOAA. By contrast, the Shannon index and the dominant taxa of AMF had no significant correlations with parameters (Figure 2J).

### The eCO_2_ promoted growth of predators and regulated their community composition

**Figure 3:**
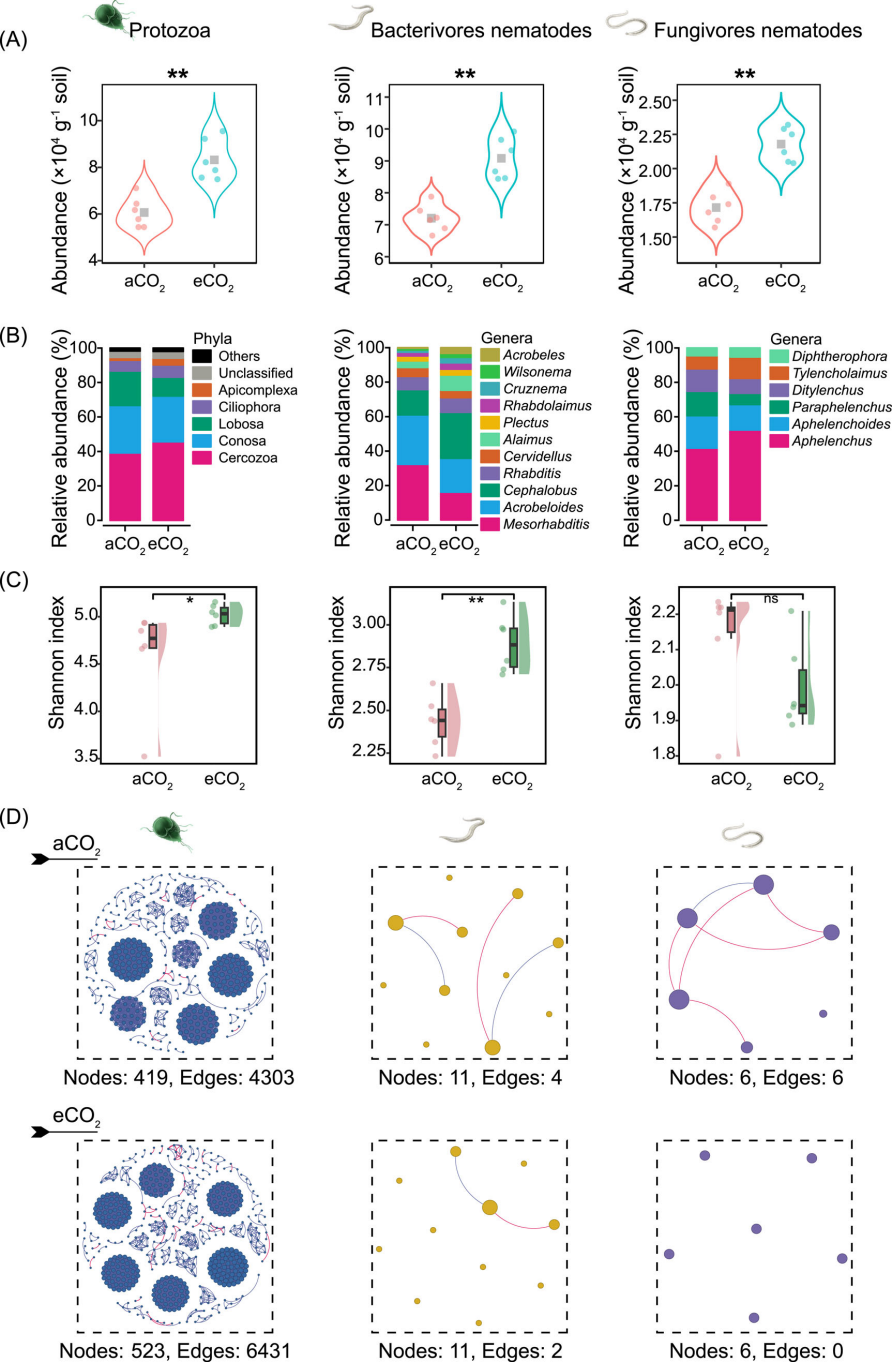
The features of predators under different CO_2_ treatments. (A–D) The abundance (A), relative abundance of taxa (B), diversity (Shannon index) (C) and co‐occurrence networks (D) of protozoa, and bacterivorous and fungivorous nematodes in the rhizosphere soil under ambient CO_2_ (aCO_2_) and elevated CO_2_ (eCO_2_). **p < 0.01; *p < 0.05. The means and standard errors (*n* = 6). ns, nonsignificant. [Correction added on 6 December 2024, after first online publication: In Figure 3D, under the eCO_2_ treatment, the values “Nodes: 23, Edges: 6431” were updated to “Nodes: 523, Edges: 6431”.]

For the predators, the eCO_2_ treatments significantly increased the abundance of protozoa by 37.2%, bacterivorous nematodes by 26.0%, and fungivorous nematodes by 27.0% (Figure 3A). Specifically, the relative abundance of dominant taxon of protozoan increased (by 16.4% for Cercozoa) and some genera of the bacterivorous nematodes decreased (*Mesorhabditis* by 50% and *Acrobeloides* by 31.4%) or increased (*Cephalobus* by 81.8%, *Alaimus* by 130.4%, *Acrobeles* by 900.0% and *Wilsonema* by 133.3%) at eCO_2_ compared with aCO_2_. Regarding the fungivorous nematodes, the eCO_2_ treatment decreased relative abundance of *Ditylenchus* by 33.3% and *Paraphelenchus* by 54.1%, whereas increased the relative abundance of *Aphelenchus* by 25.1% (Figure 3B).

The Shannon index and the community composition (the PCo1 scores) of the predator microbial community changed significantly in the eCO_2_ treatment, except for fungivorous nematodes (Figures 3C and S7). Specifically, the Shannon index of the protozoa and bacterivorous nematodes increased by 9.2% and 18.6% in eCO_2_ treatment, respectively (Figure 3C). For the protozoa community, the eCO_2_ treatment increased the nodes and edges and the average degree, whereas had no effect on connectedness. For the bacterivorous and fungivorous nematode communities, the eCO_2_ treatment decreased the edges, the average degree and connectedness (Figure 3D, Table S1).

Across the two CO_2_ environments, the abundance of protozoa, and bacterivorous and fungivorous nematodes and the Shannon index of bacterivorous nematodes had strong positive correlations with growth of AMF and ALP‐producing bacteria, wheat growth, available P, MBP, ALP activity, and TOAA exudation, but showed negative correlations with Ca‐P_i_ and NaOH‐P_o_ (Figure S8). By contrast, the Shannon index of protozoa and fungivorous nematodes showed weakly correlations with most of the parameters (Figure S8). The dominant taxa of fungivorous and bacterivorous nematodes also showed the tight correlations with plant growth traits and soil properties (Figure S6). Additionally, the community composition of bacterivorous nematodes had significant correlations with crop growth, P fractions, ALP activity, and TOAA, whereas the community composition of fungivorous nematodes had correlations only with P accumulation, ALP activity, and TOAA. The communities of protozoa did not correlate with any plant and soil parameters across the two CO_2_ environments (Figure S5).

### The eCO_2_ strengthened trophic interactions among rhizosphere microbiota

Across the two CO_2_ environments, the relative abundance of AMF dominant taxa did not correlate significantly with the relative abundance of dominant protozoan and nematode taxa (Figure S9). Importantly, the dominant taxa of ALP‐producing bacteria had strong and frequently significant correlations with their nematode predators. Specifically, the relative abundance of Rhizobiaceae was significantly and negatively correlated with the relative abundance of genera *Mesorhabditis* and *Paraphelenchus*, whereas the correlation was positive with *Cephalobus* (Figure S9).

**Figure 4:**
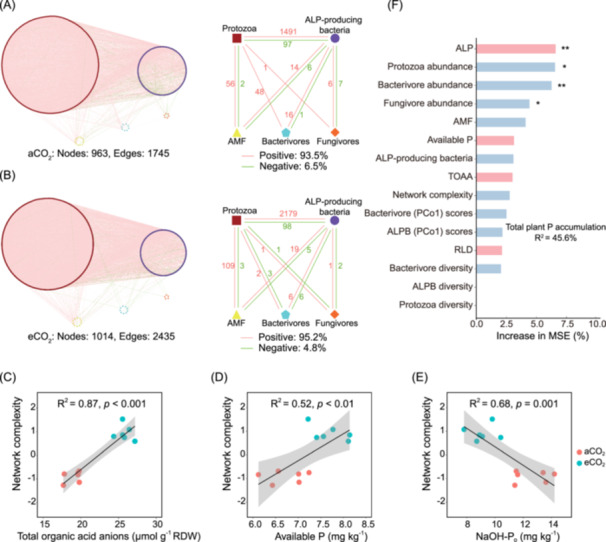
Co‐occurrence patterns of multitrophic networks in the microbiota communities under different CO_2_ treatments. (A and B) Ambient CO_2_ (aCO_2_) (A) and elevated CO_2_ (eCO_2_) (B). Each node is the taxon of AMF, ALP‐producing bacteria, protozoa, bacterivorous, or fungivorous nematodes. Size of each node is proportional to the number of connections (i.e., degree). A connection between two nodes (i.e., an edge) represents a strong (Spearman *r* > 0.6) and significant (adjusted *p* < 0.05) correlation. Pink edges indicate the positive and green edges the negative correlations between the two nodes. On the right of each network is the summary of the positive and negative edges among AMF, ALP‐producing bacteria (ALPB), protozoa, and bacterivorous and fungivorous nematodes, and the statistics for the total nodes and edges. The pink and green numbers represent the number of positive and negative edges, respectively. (C–E) Potential associations between the network complexity of the whole microbiota community and concentration of total organic acid anions (C) available P (H_2_O‐P_i_+NaHCO_3_‐P_i_) (D), and NaOH‐P_o_ (NaOH‐extractable organic P) (E) in the rhizosphere soil. (F) The random forest mean predictor importance (percentage increase in mean squared error [MSE]) of soil properties and microbial variables on soil available phosphorus concentration. The blue bars indicate microbial factors; the pink bars indicate other factors (soil P fractions, root traits). ***p < 0.01; *p < 0.05. ALP, alkaline phosphomonoesterase activity; RLD, root length density; TOAA, total organic acid anions (proxy for root exudation).

For the whole microbiota community network, the eCO_2_ treatment was associated with more complexity [a higher number of connections among co‐occurring taxa (nodes: 1014, edges: 2435) compared to aCO_2_ (nodes: 963, edges: 1745)] (Figure 4A,B). Compared with aCO_2_, in the eCO_2_ treatment the associations between ALP‐producing bacteria and protozoa (2277 edges) and AMF and protozoa (112 edges) were higher, whereas those between ALP‐producing bacteria and bacterivorous nematodes (12 edges) and protozoa and bacterivorous nematodes (5 edges) were lower (Figure 4A,B). In addition, there were positive correlations between TOAA (Figure 4C), the available P in the rhizosphere soil (Figure 4D) and the network complexity, and a negative correlation between NaOH‐P_o_ and the network complexity (Figure 4E), hinting bottom‐up effects on growth of microbiota and their trophic interactions in rhizosphere.

The random forest showed that among the factors of soil P fractions, root traits and microbiota, wheat P accumulation was significantly influenced by ALP activity, protozoa abundance, bacterivorous nematodes abundance and fungivorous nematodes abundance across the two CO_2_ environments (Figure 4F).

## DISCUSSION

We found that eCO_2_ had no impact on shoot P concentration but increased wheat shoot biomass and total P accumulation of wheat (Figure 1A). The increased P demand arising from enhanced leaf photosynthesis and plant growth under eCO_2_ can be met by a range of P acquisition strategies [35, 36]. Our results showed that eCO_2_ increased wheat root biomass and length and augmented concentration of organic acid anions in the rhizosphere soil, but had little effect on specific root length (Figure 1A), suggesting that enhanced root growth and modified physiology were critical for improving wheat P acquisition. Root exudation of organic acid anions plays a key role in P acquisition through P mobilization under eCO_2_. For instance, eCO_2_ decreased (by 24%) P uptake in the wheat genotype that lacks the capacity to exude citrate but not in the citrate‐exuding wheat genotype [37]. Similarly, Taloy et al. [36] also found that eCO_2_ enhanced P acquisition by sedges via meeting the increased carbon cost of intense exudation of P‐mobilizing compounds compared with grasses featuring relatively low root exudation. Although the eCO_2_ did not increase AMF colonization (Figure 1A), the total AMF biomass was elevated under eCO_2_ (Figure 2A) because of increased root growth (Figure 1). Reichert et al. [17] also suggested plants would benefit from investing more carbon in supporting the AMF symbioses (allowing for increased extraradical hyphae length) under eCO_2_, especially when available P concentration in soil is not too low [38].

Given that all above‐mentioned plant adaptations to increase P acquisition (i.e., root morphology, P‐mobilizing exudates, and AMF symbioses) cost photosynthetic carbon, plants generally choose the one main strategy for capturing enough P to meet shoot growth demand, with the reported tradeoffs between root morphology and exudation as well as between root morphology and AMF symbioses [39]. However, eCO_2_ may mitigate the carbon constraints through improving photosynthetic rates, which may underpin synergistic combinations of increased root length and elevated concentration of P‐mobilizing compounds in the rhizosphere soil and/or enlarged AMF biomass to enhance exploration of the soil volume for available P and promote P acquisition [4].

In our study, eCO_2_ significantly promoted total P accumulation of wheat (Figure 1A), but the available P in the rhizosphere soil was still higher under eCO_2_ than aCO_2_ (Figure 1B). This finding suggests the replenishment of the available P pool from Ca‐P_i_ and NaOH‐P_o_ fractions under eCO_2_ (Figure 1B). The high concentration of organic acid anions in the rhizosphere soil and strong activity of ALP (Figure 1) arising from the high abundance of ALP‐producing bacteria in the rhizosphere (Figure 2E) can strongly promote the mobilization of Ca‐P_i_ and the mineralization of P_o_ in alkaline soils under eCO_2_ (see also [13, 14, 23, 35, 40]). In addition, MBP in the rhizosphere soil was also significantly higher under eCO_2_ than aCO_2_ (Figure 1B). Similarly, in paddy soils under long‐term exposure to elevated CO_2_, Wang et al. reported increased microbial biomass and thus enhanced P immobilization in it, but contrary to the study presented here there was a 20% decline in available phosphorus. The competition between plants and microorganisms for P under eCO_2_ may reduce plant P uptake in P‐limited systems [18], whereby low phosphorus supply constrains plant responses to eCO_2_ [10]. However, in the intensive agricultural systems in China, the P fertilizer input is sufficient to promote crop production [41] even with the increased P immobilization in microbial biomass under eCO_2_. In addition, P immobilization in microbial biomass is generally impermanent in agriculture systems, the phosphorus can be released by microbial biomass turnover and then increase soil available P content and promote crop growth [42, 43]. Thus, understanding the form transformation of P and competition process between crops and microbes may be key for promoting productivity under future eCO_2_ scenarios.

In the present study, the elevated CO_2_ promoted the allocation of photosynthetic carbon to root biomass and exudation of organic acid anions (Figure 1), contributing to the increase in bacterial and fungal biomass (Figure S3, see also Jin et al. [23]), including ALP‐producing bacteria and AMF (Figure 2). The bacteria and fungi as basal prey resources can support energy demands of microbivores [44]. Thus, we observed a significant increase in the abundance of protozoa and bacterivorous and fungivorous nematodes (Figure 3A), suggesting a strong bottom‐up effects on the trophic cascades in the rhizosphere [45].

Compared with aCO_2_, under eCO_2_ diversity of the ALP‐producing bacteria significantly decreased (Figure 2G) and the community composition structure was significantly altered (Figure S4). This may be attributed to the abundance of substrates in the rhizosphere (such as organic acid anions) strengthening the competition among ALP‐producing bacteria under eCO_2_, with the increased relative abundance of the dominant taxa (such as Rhizobiaceae, Figure 2F) resulting in a decrease in bacterial diversity (see also Wang et al. [46]). Reducing diversity of the ALP‐producing bacteria also suggests that crop can strengthen the rhizosphere process and recruit more efficiently phosphate‐solubilizing bacteria to capture P for crop growth under eCO_2_, which also hints that eCO_2_ can promote the interaction between crop and beneficial microbes.

In our study, the varying CO_2_ treatments did not influence diversity and the community composition of AMF (Figures 2 and S4), and despite the AMF abundance strongly correlating with a number of plant and soil properties (Figure 2J), there were no correlations between diversity and the community composition of AMF and plant and soil properties (Figures 2J and S5). By contrast, Cotton et al. [47] showed that elevated CO_2_ increased the ratio of Glomeraceae to Gigasporaceae in soybean roots in three growing seasons over 5 years. However, they also found that greatest differences in the AMF community composition were not due to different CO_2_ treatments but were detected between years, suggesting a strong importance of temporal dynamics [47]. Generally, the assembly of AMF community is strongly driven by abiotic factors, such as soil pH and nutrient content, than by the host plants [25, 48]. However, in the present study, the difference of available P in rhizosphere between aCO_2_ and eCO_2_ (6.73 vs. 7.70 mg kg^−1^), albeit significant (Figure 1B) might not have been sufficient to impact the AMF community composition. Similarly, Maček et al. [49] also found that long‐term eCO_2_ significantly increased AMF abundance but had a small effect on the composition of AMF community. Overall, our findings suggested that promoting AMF biomass rather than regulating community composition may be the optimal strategy to more efficiently capture P for meet crop growth demand under eCO_2_.

The Shannon indices of protozoa and bacterivorous nematodes increased significantly under eCO_2_ (Figure 3C). This was likely due to the increased abundance of prey (bacteria) (Figure S3, see also Wardle et al. [50]), that lead to the relative abundance of rare species of protozoa and bacterivorous nematodes increased under eCO_2_ (Figure 3B). Diversity and composition of ALP‐producing bacteria and bacterivorous nematodes had more significant correlations with plant growth and soil P fractions compared to the protozoa and fungivorous nematodes under different CO_2_ environments (Figures 2, S5 and S8). Compared to the fungi and fungivorous nematodes, the bacteria and bacterivorous nematodes are considered as the r‐strategists (short life cycle and high fecundity) that can respond quickly to environmental changes [19]. The eCO_2_ promotes plant photosynthesis and root exudation of low‐molecular‐weight compounds (e.g., organic acid anions) (cf. Figure 1), which would readily influence the diversity and composition of soil biota with r‐strategy. For instance, there were tight positive correlations between the dominant ALP‐producing bacteria Rhizobiaceae or dominant bacterivorous nematodes *Cephalobus* and various plant growth and soil parameters (including concentration of organic acid anions in the rhizosphere soil) (Figure S6), implicating that selective predation may play an important role not only in shaping the community composition of ALP‐producing bacteria and bacterivorous nematodes but also in governing plant growth.

Network analysis can be applied to identify co‐occurrence patterns and potential interactions in a complex biotic community [51]. In our study, the whole multitrophic network (bacteria, fungi, protozoa, and nematodes) exhibited higher topological metrics under eCO_2_ than aCO_2_ (Table S2), indicating a highly complex network [52, 53]. In addition, the multitrophic interactions, especially the associations between protozoa and microorganisms (i.e., AMF and ALP‐producing bacteria) were significantly strengthened by eCO_2_ (Figure 4). The higher P availability and increased concentration of organic acid anions in the rhizosphere soil (Figure 1) under eCO_2_ can lead to rapid growth of microbiota across multitrophic levels through bottom‐up linkages (Figure 4), thereby enhancing the interactions between protozoa and microorganisms [54, 55]. Conversely, the associations between bacterivorous nematodes and protozoa, and between bacterivorous nematodes and ALP‐producing bacteria were weakened by increasing CO_2_, with the positive edges significantly decreased (from 64 to 8) and negative edges significantly increased (from 1 to 9) with increasing CO_2_ (Figure 4). Given that negative edges indicate the competitive relationships in the network [56], the eCO_2_ may strengthen the competition between protozoa and bacterivorous nematodes preying on ALP‐producing bacteria, which is consistent with the stress‐gradient hypothesis [57]. These findings indicate that protozoa are tightly linked within microbiomes as potential key microbiome controllers. The stronger links between protozoa and AMF as well as ALP‐producing bacteria under eCO_2_ also indicate protozoa selectively feed on bacterial and fungal prey [58, 59, 60], which may play important roles in soil P cycling in agricultural system under climate change.

## CONCLUSION

Elevated CO_2_ promoted wheat shoot growth and P concentration, which was likely due to eCO_2_: (i) promoting root length and AMF growth that would enhance the capture of available P, (ii) increasing the release of root organic acid anions associated with enhanced solubilization of Ca‐P_i_ in the rhizosphere soil, and (iii) stimulating growth of ALP‐producing bacteria that would drive organic P mineralization via high alkaline phosphomonoesterase activity. Elevated CO_2_ also strongly shaped the diversity and composition of multitrophic microbiota in the rhizosphere soil and strengthened the complex trophic interactions among AMF, ALP‐producing bacteria, protozoa, and bacterivorous and fungivorous nematodes. Future study can focus on the long‐term impacts, soil‐type variability, and the role of microbial competition for phosphorus under eCO_2_.

The findings presented here indicate the coordinated responses of root traits and rhizosphere multitrophic microbiota to eCO_2_. Based on the current knowledge, we suggest that microbiota trophic linkage can play the multifaceted and key roles in crop growth and ecosystem function under different agricultural managements and the climate change situations. The coordination of roots and multitrophic microbiota may be beneficial due to accelerated nutrient cycling [61], inhibited growth of pathogenic bacteria [27], and consequently suppressed plant diseases and improved plant health [62], based on the bottom‐up and top‐down processes. Significant structural diversity and plasticity in coordination of root and microbiota trophic interactions in the rhizosphere soil are needed to underpin ecosystem services under environmental changes.

## METHODS

### Study site and experimental design

The FACE system was established at the experimental base of Institute of Agricultural Environment and Sustainable Development, Chinese Academy of Agricultural Sciences, Changping District, Beijing (116° 8′ E, 40° 8′ N). The area had the temperate monsoon climate with mean annual temperature of 17.9°C and mean annual precipitation of 517.8 mm. The soil type is brown aquic soil. The initial soil pH in the surface layer (0–15 cm) was 8.7 (1:5, soil:water), and the contents of organic carbon, available N, and Olsen‐P were 8.18 g kg^−1^, 94.4 mg kg^−1^, and 15.8 mg kg^−1^, respectively.

The experiment was initiated in 2007 with a double cropping of wheat‐soybean rotation during 2007.06–2014.06 and wheat‐maize rotation after 2014.10, comprising two treatments: ambient CO_2_ (aCO_2_, 415 ± 16 µmol mol^−1^) and elevated CO_2_ (eCO_2_, 550 ± 17 µmol mol^−1^). The FACE system platform comprised octagonal rings 4 m in diameter. To minimize the interference between CO_2_ treatments, the distance between the rings was more than 14 m. The CO_2_ concentration in the FACE rings was controlled by a computer in real time according to the wind speed and direction. The timing of CO_2_ release (during the day but no release at night) was automatically regulated by the sunrise and sunset.

The experiment was set up in a randomized complete block design with three replications. The 75 kg N ha^−1^ (urea), 72 kg P ha^−1^ (superphosphate) and 75 kg K ha^−1^ (potassium sulfate) were applied as basal fertilizer before sowing wheat in early October. At the end of April (jointing stage of wheat), N fertilizer (urea) was applied as topdressing at 95 N kg ha^−1^.

### Sample collection and determination

The plant and soil sample were collected at the wheat jointing stage in April 2022. In each plot, two subplots were established for obtaining six replicate samples to satisfy the complex statistical analysis. In each subplot, wheat and soil samples were collected from a 20 × 20 cm area (with a plant in the center) using a spade to the depth of 15 cm. The sampling depth was chosen based on the two reasons. (1) At jointing stage, about 50% of the wheat root system was distributed in the soil layer 0–15 cm [63]. (2) The protozoa and nematodes were mainly active in the soil layer 0–15 cm [64]. All the shoot and root material in the sampling area was collected to measure plant biomass and root morphological and physiological traits. All the roots in the sampling space were carefully removed from the soil then shaken gently to remove the loosely adhering soil, leaving the tightly adhered soil defined as rhizosphere soil. A fine‐bristle brush was used to brush soil off roots to get the rhizosphere soil. After passing through a 2 mm sieve, each rhizosphere soil sample was split into three parts. One part was used for soil P fractionation, ALP activity, and microbial biomass C, N, and P measurements; the second part was kept at −80°C for phospholipid fatty acid analysis and soil genomic DNA extraction; the third part was stored at 4°C for protozoa and nematode extractions.

A subsample of roots was repeatedly dunked into the 50 mL of 0.2 mmol L^−1^ CaCl_2_ solution until as much rhizosheath soil as possible was removed, taking care to minimize root damage. Following centrifugation at 13,000 *g* for 5 min, the supernatant was filtered through a 0.22 μm syringe filter, three drops of microbial inhibitor Micropur (Sicheres Trinkwasser) were added to 10 mL of supernatant, and the samples were stored at −20°C until analysis of carboxylates (organic acid anions) using a reversed‐phase high‐performance liquid chromatography system [65]. Afterwards, the roots were frozen at −20°C until the assessment of morphology and dry weight.

After thawing, roots were washed in deionized water and scanned on an Epson flatbed scanner (V800) at the resolution of 300 dpi, and then analyzed with WinRhizo® software (Regent Instruments) to obtain root length.

About 50 root tips (1‐cm length) were randomly chosen in each sample and stained with acid fuchsin solution to quantify AMF colonization of roots under a light microscope [66]. The remaining roots were dried at 75°C until constant weight. Specific root length was calculated as root length per unit dry mass, and root length density was calculated as the root length divided by soil volume.

The wheat root and shoot samples were crushed and digested in a mixture of 5 mL of concentrated sulfuric acid and 8 mL of 30% v/v H_2_O_2_. The P concentration was analyzed by the standard vanadomolybdate method [67].

### Determination of soil P fractions and alkaline phosphomonoesterase activity

The soil total P concentration was measured by Inductively Coupled Plasma‐Atomic Emission Spectrometry (ICP‐AES; Optima 8300) after microwave digestion (HNO_3_‐H_2_O_2_‐HF). For quality assurance, the reference material from the National Quality and Technology Supervision Agency of China (GBW07446) was used.

The P fractions in the rhizosphere soil were determined by the improved Hedley phosphorus fractionation [68]. In brief, 0.5 g soil samples were fractionated to determine soil P components by successively adding different extractants. (1) Deionized water and (2) 0.5 M NaHCO_3_ (pH = 8.5) were used to determine water‐soluble P_i_ (H_2_O‐P_i_) and NaHCO_3_‐P_i_, respectively, “collectively called available P.” (3) 0.1 M NaOH was used to etract NaOH‐P_i_ and NaOH‐P_o_ representing moderately labile P (NaOH‐P_t_). (4) 1 M HCl was used to extract Ca‐P_i_ (apatites, other different calcium phosphates and P sorbed onto CaCO_3_). (5) Residual P was determined by calculation: Total P–Available P–NaOH‐P_t_–Ca‐P_i_. The Ca‐P_i_ and NaOH‐P_o_ can be transformed into available P though the mobilization and mineralization.

Microbial biomass P concentration was analyzed using the chloroform fumigation‐extraction method with 2 g of dry rhizosphere soil. The ALP activity in the rhizosphere soil was determined using *p*‐nitrophenyl (*p*‐NP) phosphate as the substrate with the buffer adjusted to pH 11.0. The ALP activity was expressed as mg *p*‐NP g^−1^ soil h^−1^.

### Lipid extraction analysis

The biomass of AMF was characterized by neutral lipid fatty acid (NLFA), whereas saprotrophic fungi and bacteria were characterized by phospholipid fatty acid (PLFA) analysis. Briefly, 2 g of freeze‐dried rhizosphere soil samples were extracted with a solution containing chloroform, methanol, and citrate buffer (1:2:0.8, v/v/v). Then, neutral, glyco‐, and phospho‐lipids were separated on a silicic acid column. The neutral lipids and phospholipids were converted to fatty acid methyl esters by mild alkaline methanolysis. Fatty acid (nonadecanoic, 19:0) was added to the prepared fatty acid methyl ester samples as an internal concentration standard to quantify phospholipids by a HP 6890 gas chromatograph (Hewlett–Packard). AMF biomass was indicated by the NLFA biomarker 16:1ω5c [69]. The PLFA biomarkers i15:0, a15:0, 15:0, i16:0, 16:1ω9, 16:1ω7t, i17:0, a17:0, cy17:0, 17:0, 18:1ω7, and cy19:0 were chosen to represent bacterial biomass, and the PLFA biomarkers 18:1ω9c and 18:2ω6,9c were used as indicators of saprotrophic fungal biomass [70]. The biomass values were expressed as nmol NLFA or PLFA g^−1^ soil.

### Illumina MiSeq sequencing and bioinformatic processing

Genomic DNA was extracted from 0.5 g rhizosphere soil using an OMEGA Soil DNA Kit (M5635‐02) (Omega Bio‐Tek) following the manufacturer's protocol. The extracted DNA was dissolved in tris‐EDTA buffer and quantified by a ND‐1000 spectrophotometer (NanoDrop Technologies). For assessment of AMF community, the primers AMV4.5NF and AMDGR were used for the amplification by qPCR [71]. Primers ALPS‐730F and ALPS‐1101R were used to amplify the bacterial *phoD* gene by qPCR [72]. Abundance of ALP‐producing bacteria was assessed using *phoD* gene copy numbers. The qPCR of protozoa was performed using the primers TAReuk454FWD1F and TAReukREV3 [73].

Purified amplicons were sequenced on an Illumina MiSeq platform (2 × 300, pair end). Sequences were quality‐filtered, denoised, merged, and chimeric sequences removed through a DADA2 plugin, after which amplicon sequencing variants (ASVs) were generated. Taxonomic identities were assigned to ASVs by the classify‐sklearn naïve Bayes taxonomy classifier in the feature‐classifier plugin [74]. The ASVs of AMF were clustered by blasting against the MaarjAM database SSU VT reference and were named as the corresponding virtual taxa (VT hereafter) [75]. The ASVs of bacterial *phoD* gene were taxonomically classified by blasting against the nucleotide sequence database [76]. The Protist Ribosomal Reference (version 4.5) was used for protozoa [77]. Alpha diversity and Bray–Curtis distances of AMF, ALP‐producing bacteria, and protozoa were calculated after rarefying all samples to the same sequencing depth.

### Enumeration of protozoa

The abundance of protozoa was determined using a modified most probable number method [78]. Briefly, 5 g of fresh rhizosphere soil was suspended in 20 mL of sterile water and shaken at 180 rpm for 25 min. Threefold dilution series with Nutrient Broth (NB; Merck) with Neff's Modified Amoebae Saline (NMAS) were prepared in 96‐well microtiter plates. The plates were incubated in the dark at 20°C for up to 9 days. The wells were examined for the presence of protozoa using a Nikon Eclipse TS100 inverted optical microscope after 3, 5, and 9 days. The numbers of the protozoan taxa were determined using an automated analysis software [79], and expressed as the number of individuals g^−1^ dry soil.

### Microbe‐feeding nematode community analysis

Nematodes were extracted from 100 g of fresh rhizosphere soil using the shallow dish method. After being killed at 60°C, they were fixed in 4% v/v formalin solution. For each sample, bacterivorous and fungivorous nematodes were selected according their feeding habits [80] and counted under microscope. The abundance was expressed as the number of individuals per gram of soil dry weight. Thereafter, at least 100 individuals for each trophic group (if available in the sample) were randomly selected and identified to the genus level using an inverted Nikon Eclipse TS100 microscope. The richness and Shannon index were calculated to assess the taxonomic diversity of bacterivorous and fungivorous nematodes.

### Data analysis

The Mann–Whitney *U* test was used to evaluate effects of the eCO_2_ on plant growth, soil P fractions, root traits, AMF biomass, and the abundance of ALP‐producing bacteria, protozoa, and bacterivorous and fungivorous nematodes in comparison to the ambient CO_2_. And, we used Spearman correlation test to examine the relationship between the plant traits and soil properties and the content of soil P fractions.

The relative abundance and α‐diversity (Shannon index) were calculated based on the rarefied abundance table of the microbiota; then, the differences between the two CO_2_ treatments were compared by the Mann–Whitney *U* test. For each of the five microbiota communities, we selected the top three groups in relative abundance as the dominant taxa, and then analyze the relationships between the dominant taxa and environmental factors, including plant growth, P fractions and root traits with the Spearman correlation coefficients. In addition, we used Spearman correlation test to examine the relationship between the AMF biomass, the abundance of ALP‐producing bacteria, protozoa, bacterivorous and fungivorous nematodes and the environmental factors, as well as between the Shannon index of five microbiota communities and the environmental variables under CO_2_ treatments.

On the basis of the relative abundance of taxa, we calculated Bray–Curtis dissimilarity matrices for AMF, ALP‐producing bacteria and protozoa using ASV data, and for nematodes using genus level data with the vegdist function in R [81]. Next, a principal coordinate analysis (PCoA) was conducted, and the PCo1 score was used as the indicator of community composition and assessed the significance of differences in taxonomic composition of microbiota communities. We clarified the relationship between microbiota community composition and the environmental factors by using Mantel test to correlate distance‐corrected dissimilarities of microbiota taxonomic community composition (rarefied abundance table) with those of the environmental variables.

We constructed the co‐occurrence networks for the corresponding communities to visualize potential interactions among individual taxa of AMF, ALP‐producing bacteria, protozoa, bacterivorous nematodes, fungivorous nematodes, and of the whole microbiota community in each CO_2_ environment. For the whole microbiota community, the relative abundance table was established by combining AMF VTs, ALP‐producing bacteria ASVs, protozoan ASVs, and the genera of bacterivorous and fungivorous nematodes. In the network, interactions were identified by Spearman's correlations with a threshold of *p* < 0.05 and correlation coefficients |r| > 0.6; then, the *p* value was adjusted by the Benjamini–Hochberg procedure to reduce the chances of obtaining false‐positive results. Visualization was performed using Gephi (versions 10.1) software.

To evaluate the network complexity, we calculated the topological parameters: node and edge numbers, average degree, and connectedness of the AMF, ALP‐producing bacteria, protozoa, and bacterivorous and fungivorous nematodes. We extracted subnetworks of the whole microbiota community network by preserving the phylotypes of individual soil samples using the “induced‐subgraph” function in the “igraph” package [82]. The first principal component of the topological parameters, including node and edge numbers, average degree, and connectedness, was calculated for the network complexity.

To evaluated the contribution of different factors (soil P fractions, root traits, and microbe) to the wheat P accumulation, we used the random forest analysis in the “randomForest” package in R [83].

All statistical analyses were performed using R version 4.1.2.

## AUTHOR CONTRIBUTIONS

**Na Zhou**: Writing—original draft; data curation; formal analysis; visualization. **Xue Han**: Software; investigation; project administration; validation. **Ning Hu**: Data curation; investigation; validation. **Shuo Han**: Formal analysis; visualization. **Meng Yuan**: Formal analysis; visualization. **Zhongfang Li**: Supervision. **Sujuan Wang**: Investigation. **Yingchun Li**: Investigation. **Hongbo Li**: Writing—review and editing; funding acquisition; resources; conceptualization. **Zed Rengel**: Writing—review and editing; methodology. **Yuji Jiang**: Methodology; writing—review and editing; conceptualization. **Yilai Lou**: Writing—review and editing; methodology; software; conceptualization.

## CONFLICT OF INTEREST STATEMENT

The authors declare no conflict of interest.

## ETHICS STATEMENT

No animals or humans were involved in this study.

## Supporting information


**Figure S1:** The content of P fraction in the rhizosphere soil under different CO_2_ treatments.
**Figure S2:** The relationship between the wheat growth and root traits and soil P fractions.
**Figure S3:** The biomass of microbes in the rhizosphere soil under ambient CO_2_ (aCO_2_) and elevated CO_2_ (eCO_2_).
**Figure S4:** The first principal coordination (PCo1) scores of microbial communities in the rhizosphere soil under ambient CO_2_ (aCO_2_) and elevated CO_2_ (eCO_2_).
**Figure S5:** Correlations between the composition of each soil microbiota community (AMF, protozoa, bacterivorous and fungivorous nematodes) and plant traits and soil properties.
**Figure S6:** The relationship between the relative abundance of the dominant taxa of the AMF, ALP‐producing bacteria, protozoa, and bacterivorous and fungivorous nematode communities and the environmental factors.
**Figure S7:** The first principal coordination (PCo1) scores of microbial communities in the rhizosphere soil under different CO_2_ treatments.
**Figure S8:** The relationship between the abundance and the Shannon index of soil microbiota communities (protozoa, bacterivorous and fungivorous nematodes) and plant traits and soil properties.
**Figure S9:** The correlation between the abundance of dominant taxa in AMF or ALP‐producing bacteria and the abundance of dominant taxa in the communities of protozoa or bacterivorous or fungivorous nematodes.

**Table S1:** Properties of the co‐occurrence networks of AMF, ALP‐producing bacteria, protozoa, and bacterivorous and fungivorous nematodes obtained under different CO_2_ treatments.
**Table S2:** Network complexity indices (means ± SE) of the co‐occurrence networks of whole microbiota community under different CO_2_ treatments.

## Data Availability

The raw sequence data reported in this paper have been deposited in the Genome Sequence Archive in BIG Data Center, Beijing Institute of Genomics (BIG), Chinese Academy of Sciences, under accession numbers CRA018024, CRA018025, and CRA018026 that are publicly accessible at http://ngdc.cncb.ac.cn/gsa. The data and scripts used are saved in GitHub http://github.com/xhhhhhhhhhhx/2024imate_zhou. Supporting Information (figures, tables, graphical abstract, slides, videos, Chinese translated version, and update materials) may be found in the online DOI or iMeta Science http://www.imeta.science/.
