# Supplementary material for: The crop mined phosphorus nutrition via modifying root traits and rhizosphere micro‐food web to meet the increased growth demand under elevated CO2
Source: Imeta. 2024 Oct 25;3(6):e245. doi: 10.1002/imt2.245 (PMC11683460; doi:10.1002/imt2.245)
Supplement: Supplementary file 1 — Figure S1: The content of P fraction in the rhizosphere soil under different CO2 treatments. Figure S2: The relationship between the wheat growth and root traits and soil P fractions. Figure S3: The biomass of microbes in the rhizosphere soil under ambient CO2 (aCO2) and elevated CO2 (eCO2). Figure S4: The first principal coordination (PCo1) scores of microbial communities in the rhizosphere soil under ambient CO2 (aCO2) and elevated CO2 (eCO2). Figure S5: Correlations between the composition of each soil microbiota community (AMF, protozoa, bacterivorous and fungivorous nematodes) and plant traits and soil properties. Figure S6: The relationship between the relative abundance of the dominant taxa of the AMF, ALP‐producing bacteria, protozoa, and bacterivorous and fungivorous nematode communities and the environmental factors. Figure S7: The first principal coordination (PCo1) scores of microbial communities in the rhizosphere soil under different CO2 treatments. Figure S8: The relationship between the abundance and the Shannon index of soil microbiota communities (protozoa, bacterivorous and fungivorous nematodes) and plant traits and soil properties. Figure S9: The correlation between the abundance of dominant taxa in AMF or ALP‐producing bacteria and the abundance of dominant taxa in the communities of protozoa or bacterivorous or fungivorous nematodes. [file IMT2-3-e245-s001.docx]

**Supporting information to**

**The crop mined phosphorus nutrition via modifying root traits and rhizosphere micro-food web to meet the increased growth demand under elevated CO_2_**

**Running title**: Root- and microbiota-mediated P dynamics under elevated CO_2_

Na Zhou^1^, Xue Han^1^, Ning Hu^2^, Shuo Han^1^, Meng Yuan^1^, Zhongfang Li^2^, Sujuan Wang^2^, Yingchun Li^1^, Hongbo Li^1*^, Zed Rengel^3^, Yuji Jiang^4*^, Yilai Lou^1*^

^1^Institute of Environment and Sustainable Development in Agriculture, Chinese Academy of Agricultural Sciences, Beijing 100081, China

^2^School of Food and Biological Engineering, Hezhou University, Hezhou 542899, China

^3^Soil Science & Plant Nutrition, UWA School of Agriculture and Environment, The University of Western Australia, Perth 6009, Australia

^4^College of Resources and Environment, Fujian Agriculture and Forestry University, Fuzhou 350002, China

***Corresponding address:** lihongbo@caas.cn (Hongbo Li), [yjjiang@issas.ac.cn](mailto:yjjiang@issas.ac.cn) (Yuji Jiang), [louyilai@caas.cn](mailto:louyilai@caas.cn) (Yilai Lou)

**Supplementary figures**

**
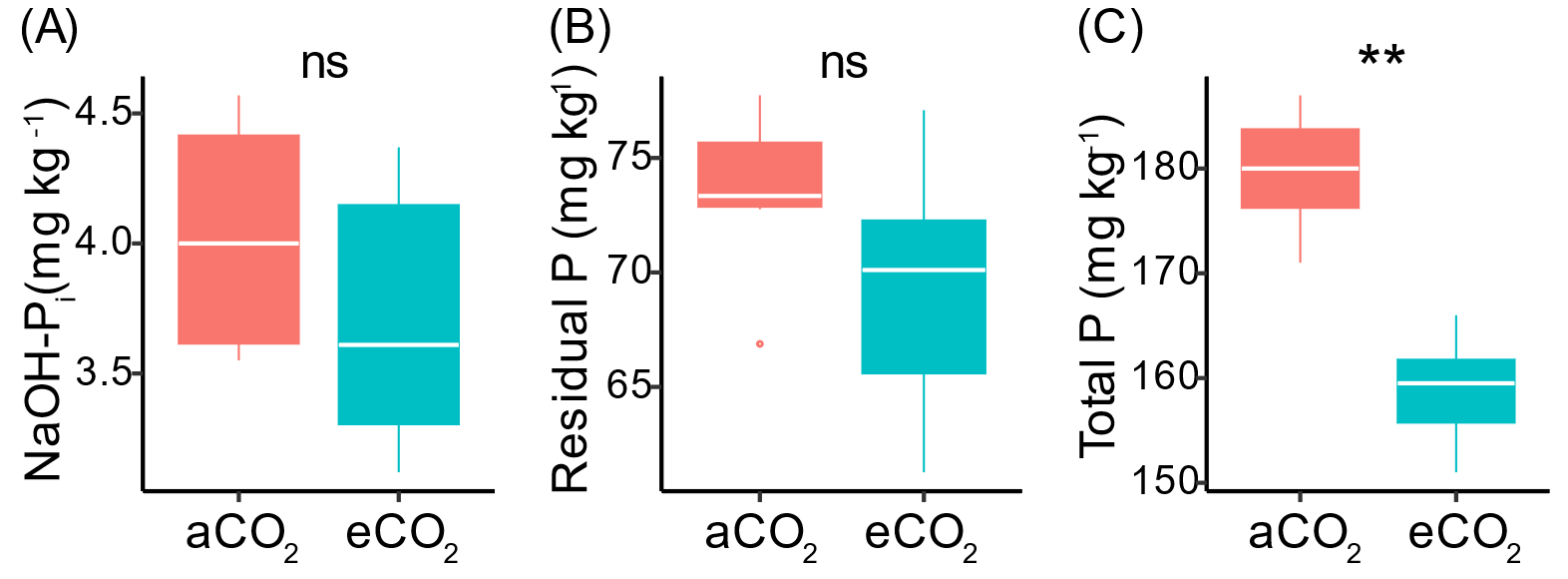
**

**Figure S1** **The content of P fraction in the rhizosphere soil under different CO_2_ treatments.** (A) NaOH-P_i_, (B) residual P, (C) total P. Means ± SE (*n* = 6). ** *p*  <  0.01; ns, non-significant.


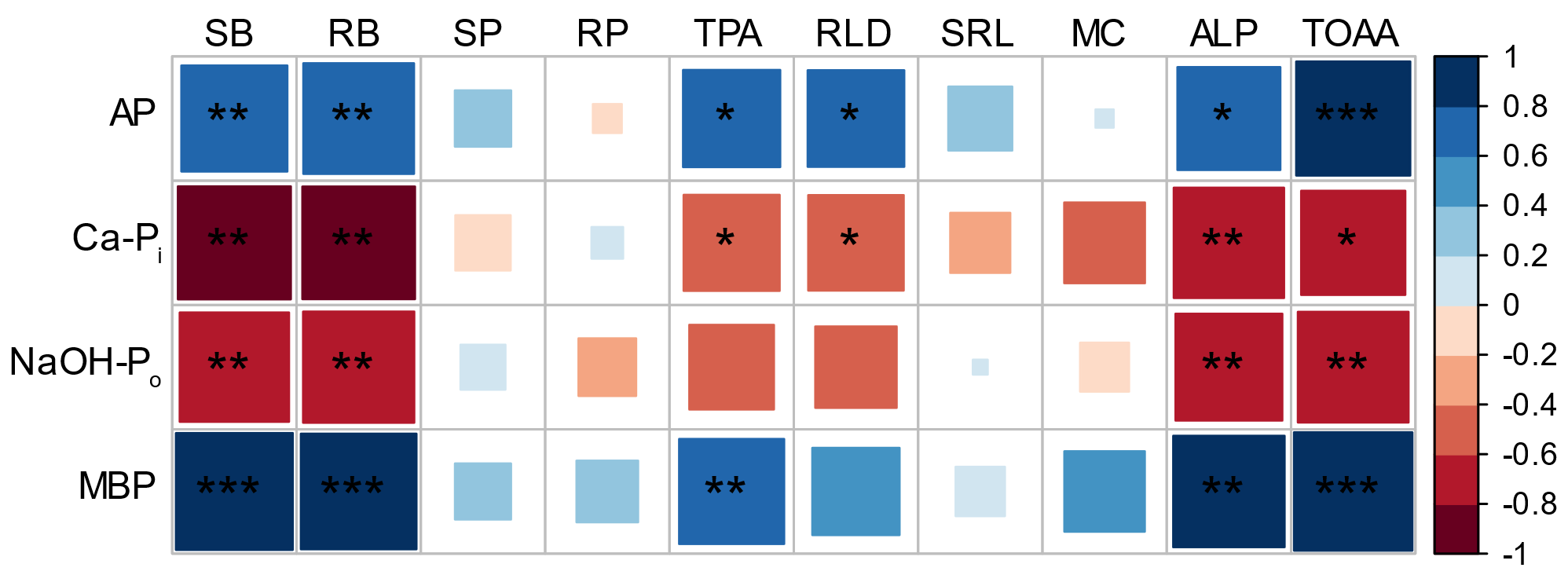


**Figure S2 The relationship between the wheat growth and root traits and soil P fractions.** SB, shoot biomass; RB, root biomass; SP, shoot P concentration; RB, root P concentration; TPA, total plant P accumulation; RLD, root length density; SRL, specific root length; MC, root mycorrhizal colonization; ALP, alkaline phosphomonoesterase activity; TOAA, total organic acid anions. AP, available P in the rhizosphere soil; Ca-P_i_, HCl-extractable inorganic P; NaOH-P_o_, NaOH-extractable organic P; MBP, microbial biomass P. *** *p*  <  0.001; ** *p*  <  0.01; * *p*  <  0.05.


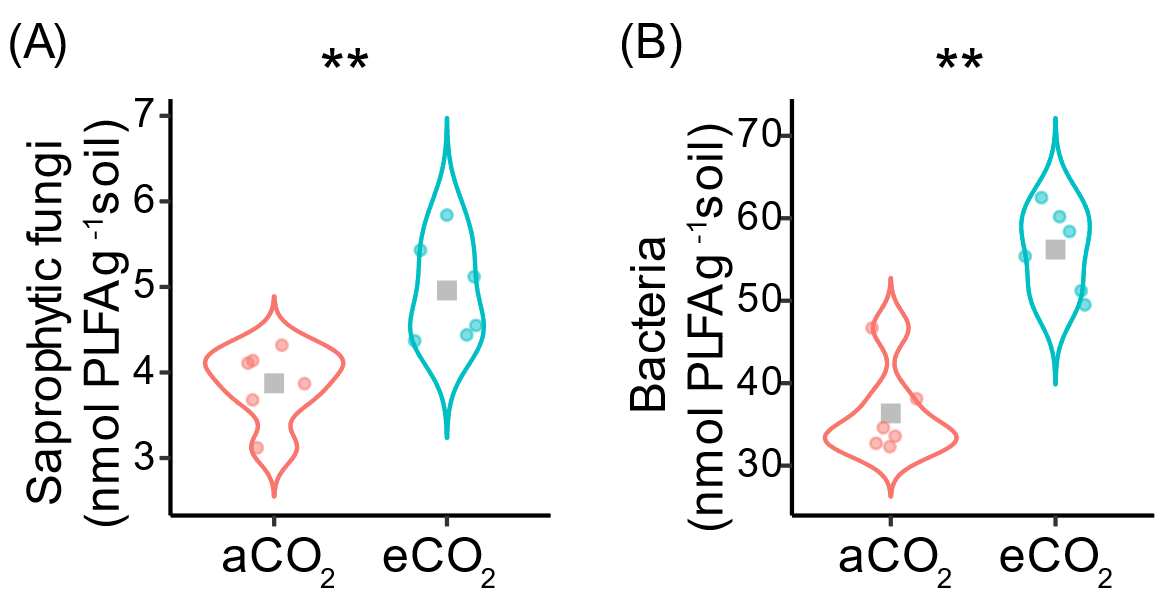


**Figure S3** **The biomass of microbes in the rhizosphere soil under ambient CO_2_ (aCO_2_) and elevated CO_2_ (eCO_2_)**. (A) saprophytic fungi, (B) bacteria. PLFA, phospholipid fatty acid. Bars represent standard errors (*n* = 6). ** *p*  <  0.01.

**
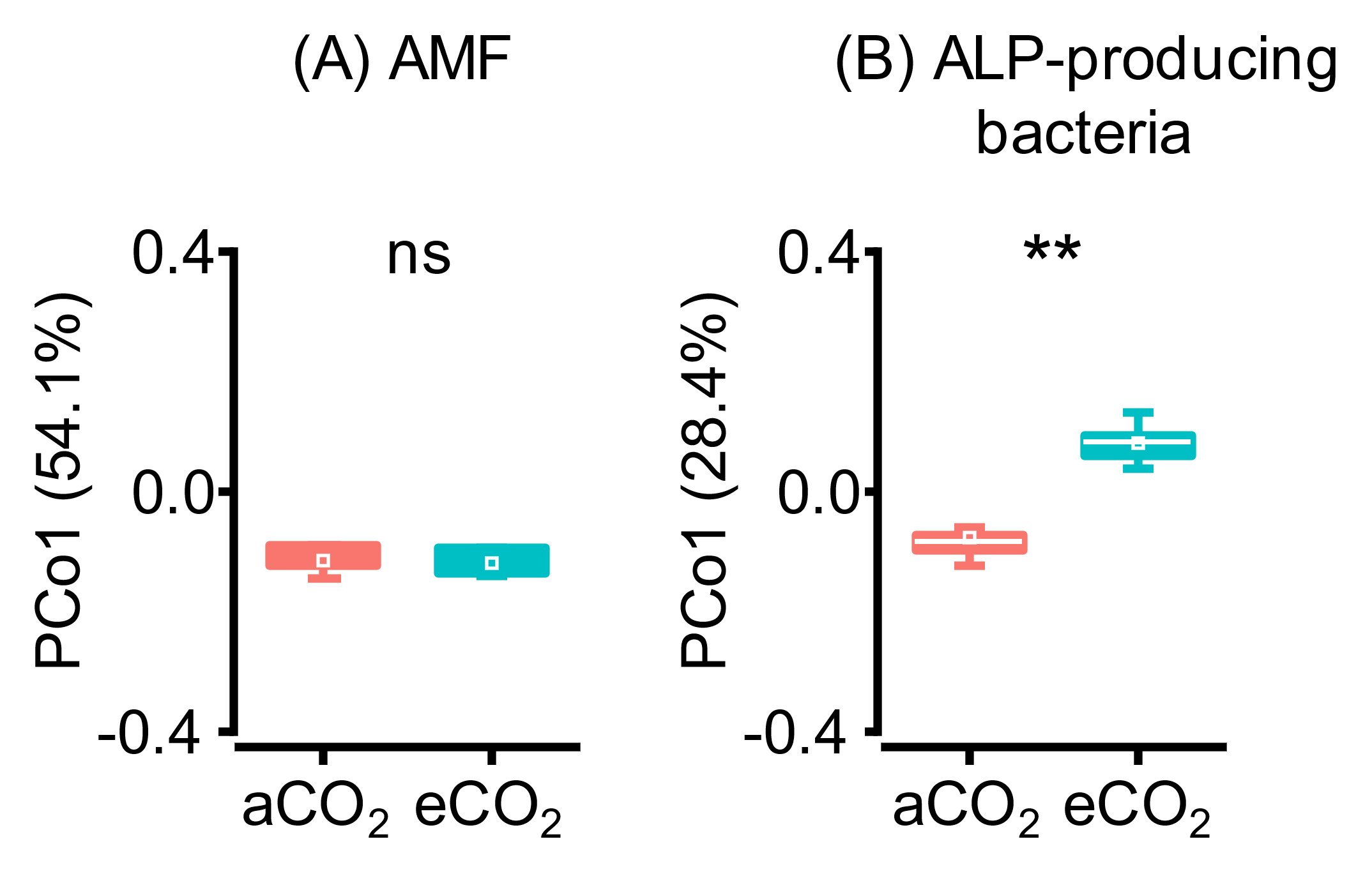
**

**Figure S4 The first principal coordination (PCo1) scores of** **microbial** **communities in the rhizosphere soil under ambient CO_2_ (aCO_2_) and elevated CO_2_ (eCO_2_).** (A) AMF, Arbuscular mycorrhizal fungi; (B) ALP-producing bacteria, alkaline phosphomonoesterase-producing bacteria. ** *p*  <  0.01; ns, non-significant.


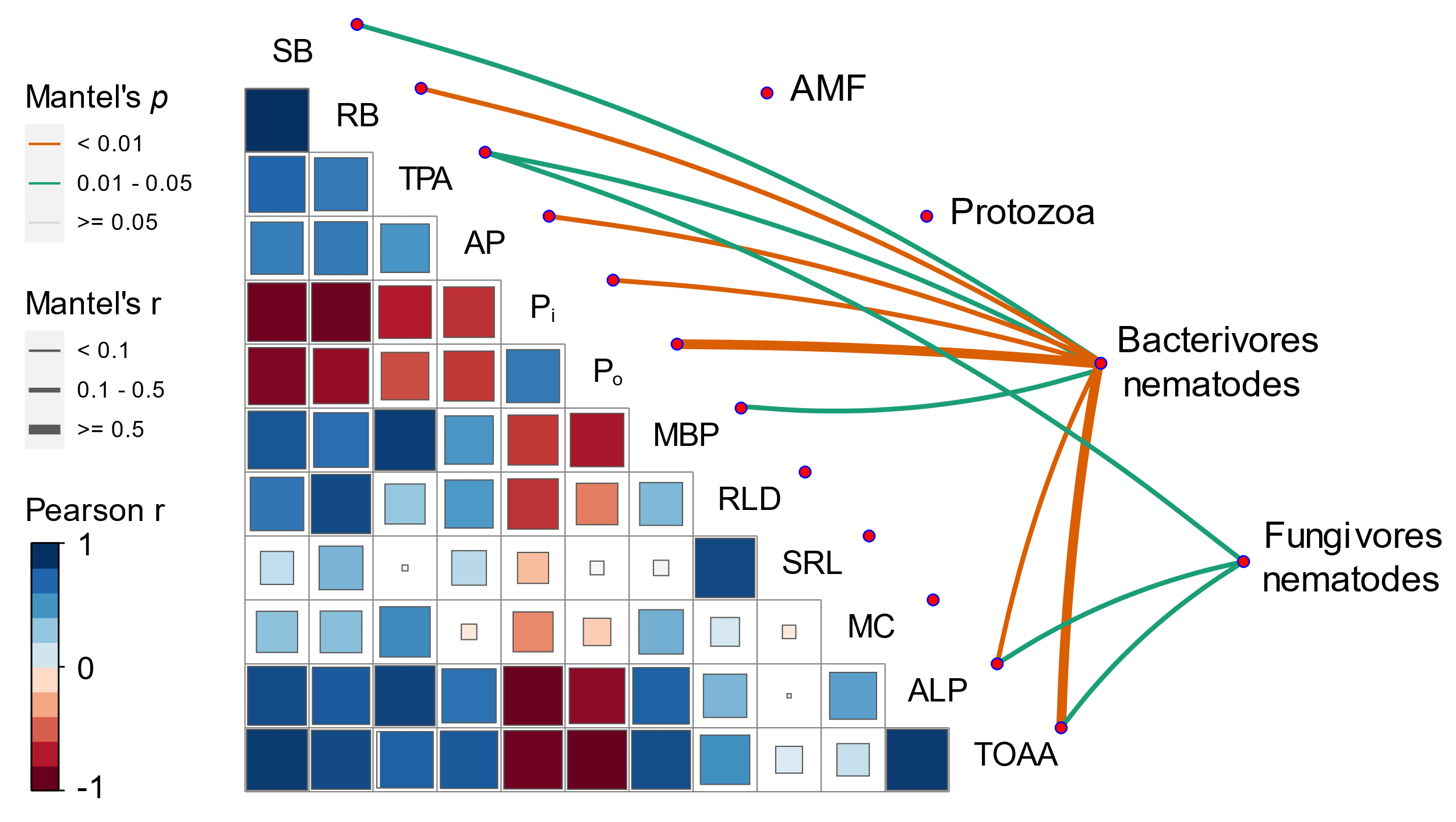


**Figure S5 Correlations between the composition of each soil microbiota community (AMF, protozoa, bacterivorous and fungivorous nematodes) and plant traits and soil properties.** Pair-wise comparisons of those traits are shown, with colour gradients denoting Pearson correlation coefﬁcients. Edge width corresponds to the Mantel *r* statistic for the specific distance correlations. SB, shoot biomass; RB, root biomass; TPA, total plant P accumulation; AP, available P in the rhizosphere soil; Ca-P_i,_ HCl-extractable inorganic P; NaOH-P_o,_ NaOH-extractable organic P; MBP, microbial biomass P; RLD, root length density; SRL, specific root length; MC, root mycorrhizal colonization; ALP, alkaline phosphomonoesterase activity; TOAA, total organic acid anions.


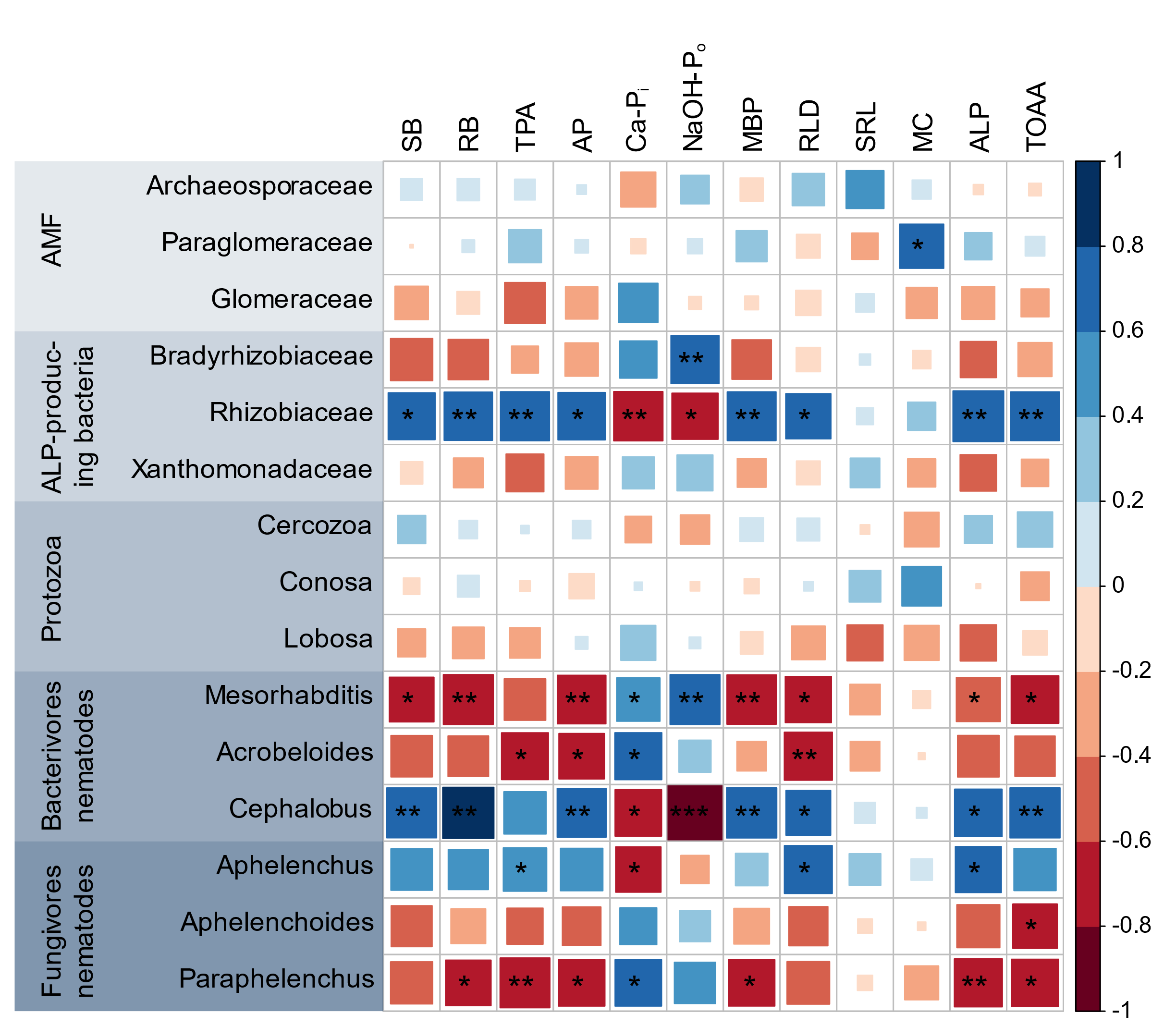


**Figure S6 The relationship between the relative abundance of the dominant taxa of the AMF, ALP-producing bacteria, protozoa, and bacterivorous and fungivorous nematode communities and the environmental factors.** SB, shoot biomass; RB, root biomass; TPA, total plant P accumulation; AP, available P in the rhizosphere soil; Ca-P_i,_ HCl-extractable inorganic P; NaOH-P_o,_ NaOH-extractable organic P; MBP, microbial biomass P; RLD, root length density; SRL, specific root length; MC, root mycorrhizal colonization; ALP, alkaline phosphomonoesterase activity; TOAA, total organic acid anions. *** *p*  <  0.001; ** *p*  <  0.01; * *p*  <  0.05.


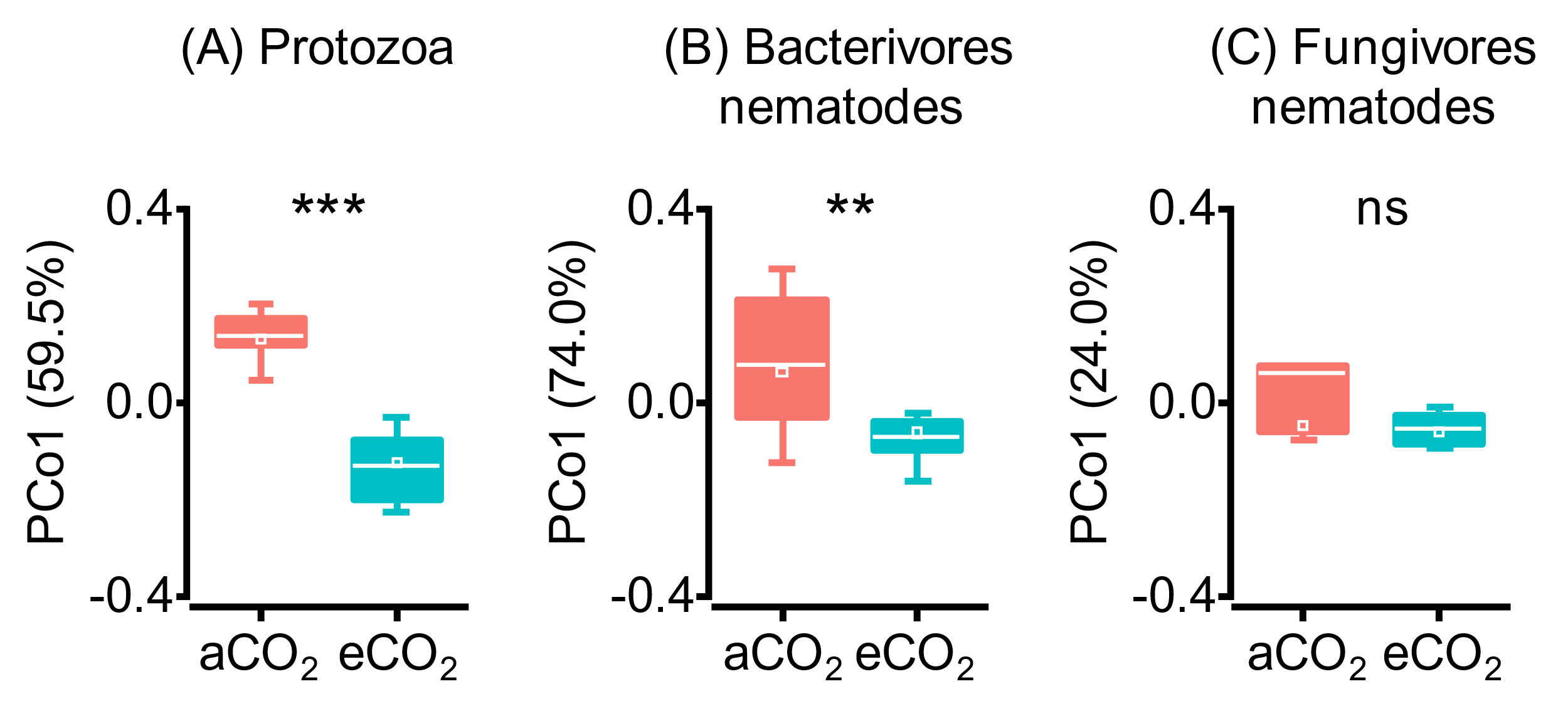


**Figure S7** **The first principal coordination (PCo1) scores of microbial** **communities in the rhizosphere soil under different CO2 treatments.** (A) protozoa, (B) bacterivorous and (C) fungivorous nematode. *** *p*  <  0.001; ** *p*  <  0.01; ns, non-significant.


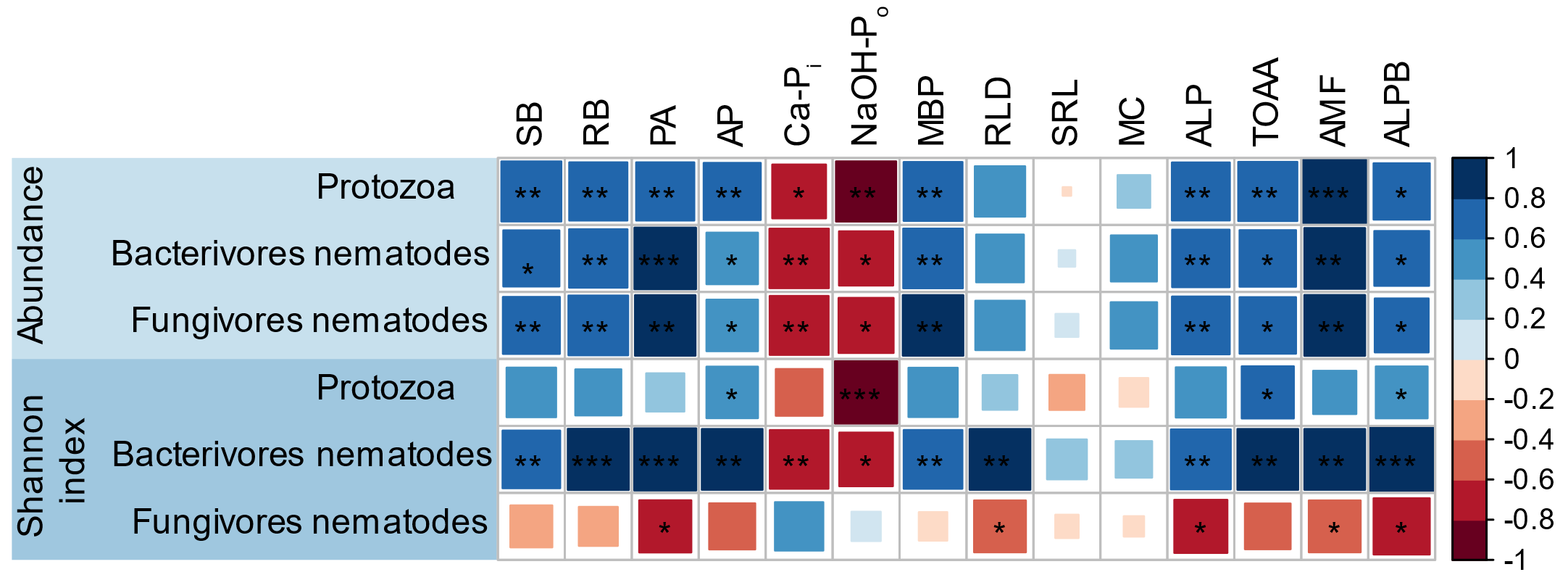


**Figure S8 The relationship between the abundance and the Shannon index of soil microbiota communities (protozoa, bacterivorous and fungivorous nematodes) and plant traits and** **soil properties.** SB, shoot biomass; RB, root biomass; TPA, total plant P accumulation; AP, available P in the rhizosphere soil; Ca-P_i,_ HCl-extractable inorganic P; NaOH-P_o,_ NaOH-extractable organic P; MBP, microbial biomass P; RLD, root length density; SRL, specific root length; MC, root mycorrhizal colonization; ALP, alkaline phosphomonoesterase activity; TOAA, total organic acid anions. AMF, AMF biomass. ALPB, ALPB abundance. *** *p*  <  0.001; ** *p*  <  0.01; * *p*  <  0.05.


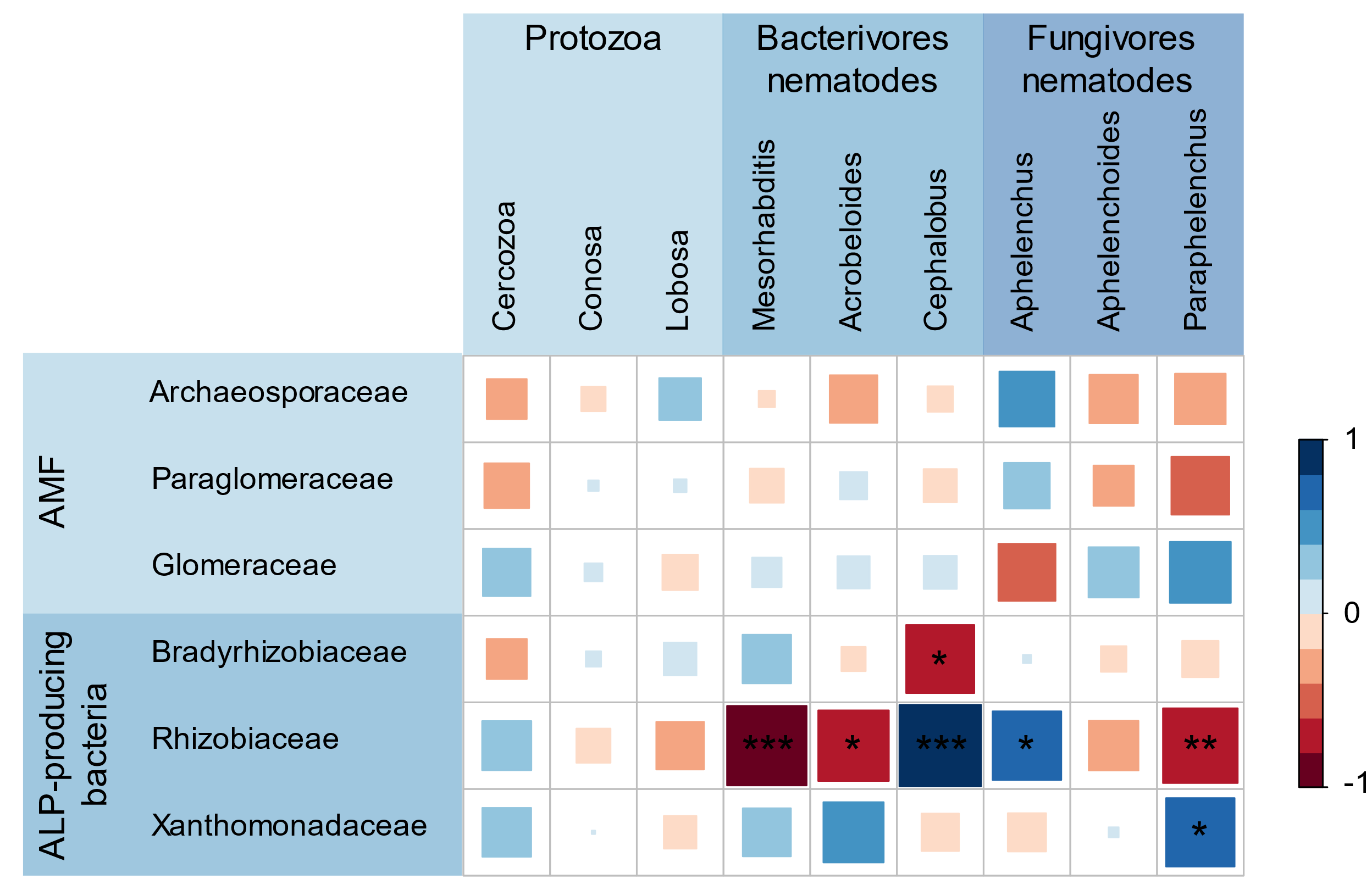


**Figure S9 The correlation between the abundance of dominant taxa in AMF or ALP-producing bacteria and the abundance of dominant taxa in the communities of protozoa or bacterivorous or fungivorous nematodes.** *** *p*  <  0.001; ** *p*  <  0.01; * *p*  <  0.05.
